# Fluorescence microscopy imaging of a neurotransmitter receptor and its cell membrane lipid milieu

**DOI:** 10.3389/fmolb.2022.1014659

**Published:** 2022-11-28

**Authors:** Francisco J. Barrantes

**Affiliations:** Biomedical Research Institute (BIOMED), Catholic University of Argentina (UCA)–National Scientific and Technical Research Council (CONICET), Buenos Aires, Argentina

**Keywords:** plasma membrane, lipid-protein interactions, cholesterol, fluorescence microscopy, single-molecule, nanoscopy, nicotinic acetylcholine receptor

## Abstract

Hampered by the diffraction phenomenon, as expressed in 1873 by Abbe, applications of optical microscopy to image biological structures were for a long time limited to resolutions above the ∼200 nm barrier and restricted to the observation of stained specimens. The introduction of fluorescence was a game changer, and since its inception it became the gold standard technique in biological microscopy. The plasma membrane is a tenuous envelope of 4 nm–10 nm in thickness surrounding the cell. Because of its highly versatile spectroscopic properties and availability of suitable instrumentation, fluorescence techniques epitomize the current approach to study this delicate structure and its molecular constituents. The wide spectral range covered by fluorescence, intimately linked to the availability of appropriate intrinsic and extrinsic probes, provides the ability to dissect membrane constituents at the molecular scale in the spatial domain. In addition, the time resolution capabilities of fluorescence methods provide complementary high precision for studying the behavior of membrane molecules in the time domain. This review illustrates the value of various fluorescence techniques to extract information on the topography and motion of plasma membrane receptors. To this end I resort to a paradigmatic membrane-bound neurotransmitter receptor, the nicotinic acetylcholine receptor (nAChR). The structural and dynamic picture emerging from studies of this prototypic pentameric ligand-gated ion channel can be extrapolated not only to other members of this superfamily of ion channels but to other membrane-bound proteins. I also briefly discuss the various emerging techniques in the field of biomembrane labeling with new organic chemistry strategies oriented to applications in fluorescence nanoscopy, the form of fluorescence microscopy that is expanding the depth and scope of interrogation of membrane-associated phenomena.

## 1 Introduction

The ultimate aim in the application of biophysical methods towards understanding the structure and function of the cell is to interrogate it with minimal perturbation. Fluorescence microscopy fulfills several criteria towards this aim: improved fluorophores and thoughtful choice of fluorescent probes maximizes cell viability by exploiting regions of the spectrum with minimal excitation-induced photodamage; high-affinity labeling provides additional minimization of invasiveness by selectively tagging subsets of molecules; new developments in instrumentation provide optimized low-damage illumination and detection conditions. These improvements have converged to push the limits of spatial resolution down to single-molecule localization and allowed the tracing of molecular motions with high temporal accuracy. Fluorescence microscopy has thus been able to delve into the structure and dynamics of membrane molecular constituents to learn about their distribution (e.g., random/non-random), orientation relative to the membrane plane, translational and rotational mobilities, and order/disorder of transient supramolecular assemblies like the ordered (“lipid rafts”)/disorder membrane lipid domains. Perhaps of greater physiological importance, fluorescence microscopy has enabled the biophysicist to gain a better understanding of the dynamic changes in these physical properties in response to a variety of cell-surface phenomena.

Among these, signaling is one of the most important and functionally relevant events occurring at the plasma membrane. The chain of events initiated by hormones or neurotransmitters at the plasmalemma is an early phylogenetic acquisition that provided the means to convey signals from the “external world” to the cell’s interior. In most metazoa this connection between the extracellular space (minimal distances for neurotransmitters; much more far-off for hormones) and the cell surface has now been established; sensory organs have either maintained the primordial link (smell or taste receptor cells sensing external chemicals from the “external world”) or developed more complicated cellular chains (sight, hearing, touch).

Chemical signaling gave rise to the appearance of membrane-bound receptors more than 4,000 million years ago, as exemplified by the superfamily of pentameric ligand-gated ion channels (pLGICs) (Ortells et al., 1992; Barrantes, 2015). The pLGICs comprise various families of neurotransmitter receptors such as γ-aminobutyric acid (GABA) type A or C receptors, the nicotinic acetylcholine receptors (nAChRs), glycine receptors, subtype 3 of the serotonin receptor family, and the structurally related glutamate-gated chloride channel (GluCl) (Zoli et al., 2018). pLGICs share a common architectural design -a pseudo-symmetric pentagonal arrangement formed by five polypeptide subunits organized around the central ion-conducting pore. The structural resemblance is related to the evolutionary history and conservation of these membrane-bound proteins (Stroud and Finer-Moore, 1985; Le Novère and Changeux, 1995; Ortells, 1998; Barrantes, 2015; Tessier et al., 2017).

In the case of the nAChRs, seventeen different subunits (α1–α10, β1–β4, γ, *ε*, and δ) have been identified to date in this family of receptors in vertebrates. These subunits can assemble into homo- or hetero-pentameric structures (Zoli et al., 2018). All subunits consist of an extracellular domain, three hydrophobic concentric transmembrane rings around the pore (Barrantes, 2003) and a relatively small extracellular carboxy-terminal domain (Karlin, 2002). In adult muscle, synaptic nAChRs are packed at very high densities (∼10,000–20,000 particles μm^−2^) at the neuromuscular junction, a very large peripheral synapse containing in the order of 10^7^ nAChR molecules (Albuquerque et al., 1974; Fertuck and Salpeter, 1976; Land et al., 1977), reviewed in (Barrantes, 1979). The actual number of receptors in the synaptic region results from the homeostatic equilibrium between synthesis, recycling and degradation of cell-surface receptors. Removal of synaptic receptors occurs *via* endocytic internalization (Engel and Fumagalli, 1982; Kumari et al., 2008). Dysregulation of these processes occurs in various neuromuscular diseases, including myasthenia gravis. In the central nervous system, heteromeric α4β2 and homomeric α7 nAChRs constitute the most abundant subtypes of nAChRs, whereas other combinations are less common, are present in smaller numbers, and usually exhibit a more limited anatomical distribution, restricted to specific brain regions (Taly et al., 2009; Zoli et al., 2018). Perturbations in cholinergic signaling in brain can affect attention, memory, cognition, and social behavior, and may be associated with major neurological and neuropsychiatric disorders, like Alzheimer disease (Barrantes et al., 2010; Chen and Mobley, 2019), Parkinson disease (Perez-Lloret and Barrantes, 2016; McKinley et al., 2019), some variants of the schizophrenia spectrum disorder (Higley and Picciotto, 2014; Caton et al., 2020), or the autistic spectrum disorder (Vallés and Barrantes, 2021).

The spatial organization of membrane proteins is closely related to their functional properties. This structure-function coupling is particularly relevant in the case of ligand-gated neurotransmitter and hormone receptors, where the signaling efficacy of the triggering ligand is strongly associated with the supramolecular organization of the receptor molecules. In this review, I illustrate the use of fluorescence microscopy and in some cases complementary “cuvette” fluorescence experiments using isolated plasma membrane samples to study membrane structure and dynamics. The contributions of fluorescence spectroscopy and fluorescence microscopy to our knowledge of biological membranes extend now over several decades. Together, they still count among the most powerful and less detrimental methods available.

## 2 Exploring the physico-chemical properties of the nAChR lipid milieu

Both neuronal-type and muscle-type nAChRs are modulated by cholesterol, making this neutral lipid the most important in lipid-receptor crosstalk. The α7 neuronal nAChR localizes in cholesterol-rich liquid-ordered lipid domains at the surface of the somatic spines in chick ciliary ganglion sympathetic neurons (Brusés et al., 2001; Marchand et al., 2002; Oshikawa et al., 2003; Zhu et al., 2006). Long-term inhibition of cholesterol biosynthesis by the statin lovastatin differentially augments cell-surface levels of α4β2 and α7 nAChRs in neurites and soma of rat hippocampal neurons (Borroni et al., 2020). Dysfunctional brain cholesterol homeostasis may therefore impact on the number of glutamate receptors at dendritic spines through cholinergic signaling involving α4β2 and α7 nAChRs. Some of the modulatory effects exerted by cholesterol on the topography and functional aspects of the nicotinic receptor are also observed with other members of the fast ligand-gated ion channels. The GABA and the glycine receptors (Bennett and Simmonds, 1996; Hénin et al., 2014; Brannigan, 2017; Lee, 2021) and the benzodiazepine receptor (Jamin et al., 2005) illustrate this point. The lessons gained from the successes and failures in the exploration of the nAChR therefore provide useful guides for the study of other neurotransmitter receptors.

### 2.1 Fluorescent cholesterol sensors and membrane probes

Given the important influence of cholesterol on nicotinic receptor function, the crosstalk between this neutral lipid and the nAChR has been the focus of numerous experimental studies (Criado et al., 1982a; Baenziger et al., 1999; Wenz and Barrantes, 2003; Baenziger et al., 2008) and see reviews on this specifc topic in (Barrantes, 2007, 2010; Barrantes, 2015; Henault et al., 2019). It is thus not surprising that fluorescence spectroscopy and fluorescence microscopy studies of the native biomembranes harboring the receptor have played a major role in elucidating some aspects of these interactions.

The study of biomembrane properties using fluorescence techniques is intertwined with the parallel development of appropriate fluorescent sensors. One of the forefathers of biological fluorescence, Gregorio Weber, was early involved in the introduction of probes to this end. This incursion into the membrane field stemmed from his more general interest in fluorescence polarization theory and applications (Weber, 1952a; b). Among the earliest examples of design organic synthesis and application of the membrane probes is the introduction of perylene and 2-methylantracene by Meir Shinitzky -then a postdoc in Weber’s laboratory- to study the microviscosity and order of the hydrocarbon region of synthetic micelles. The behavior of the fluorescent probes was analyzed using Perrin’s polarization equations (Shinitzky et al., 1971). The approach was followed by researchers in the field of biological membranes to study thermotropic phase transitions and the effect of cholesterol on the microviscosity and order of phospholipid-containing synthetic membranes (Cogan et al., 1973; Jacobson and Wobschall, 1974).

One of Gregorio Weber’s canonical probes is dansyl chloride [5-(dimethylamino) naphthalene-1-sulfonyl chloride], which he introduced in the field of fluorescence spectroscopy to study proteins in solution (Weber, 1952b). Applications in the membrane field can be found in the use of dansyl derivatives of cholesterol like 6-dansyl-cholestanol. This sensor was used to follow the trafficking of cholesterol from the plasma membrane to intracellular compartments (Wiegand et al., 2003). Friedhelm Schroeder and coworkers later focused on the physico-chemical properties of the probe in biological membranes, showing that it was mainly partitioned in cholesterol-rich liquid-ordered (Lo) domains with lower fluorescence polarization (0.20) than in cholesterol-poor, liquid-disordered (Ld) membrane regions (0.24) (Huang et al., 2010).

Another fluorescent cholesterol analogue, 6-rhodamine-cholestanol, results from the covalent coupling of a sulforhodamine group at position 6 of the sterol backbone (Maiwald et al., 2017). The probe partitions preferentially into Ld membrane domains, as measured by fluorescence microscopy of giant unilamellar vesicles (GUVs). The probe has a free 3′OH group and can thus be esterified inside the cell. Unlike 6-dansyl-cholestanol (Huang et al., 2010), 6-rhodamine-cholestanol does not follow the cholesterol storage pathway; it is internalized through endosomes and is subsequently directed to lysosomes and peroxisomes (Maiwald et al., 2017).

Robert Bittman and colleagues introduced the cholesterol probe 23-dipyrrometheneboron difluoride-24-norcholesterol (Li et al., 2006; Li and Bittman, 2007), also known as BODIPY-cholesterol, a high quantum yield cholesterol analogue that has been used to study membrane lipid order and sterol partition properties in mixed lipid phases (Marks et al., 2008), GUVs containing distinct coexisting lipid phases (Ariola et al., 2009), and living cells (Hölttä-Vuori et al., 2008; Solanko et al., 2013; Wüstner et al., 2016). Two-photon fluorescence microscopy, fluorescence lifetime and fluorescence correlation measurements were used to this end. The probe exhibits differences in translational/rotational diffusion coefficients, fluorescence correlation spectroscopy parameters, and fluorescence lifetimes between Lo and Ld phases -the latter attributed to Förster resonance energy transfer between BODIPY-cholesterol and DiI-C (12) (1,1′-didodecyl-3,3,3′,3- tetramethyl-indocarbocyanine perchlorate) (Ariola et al., 2009). BODIPY-cholesterol has since been employed as a probe with preferential partitioning in the Lo phase. Another cholesterol BODIPY probe is B-P-cholesterol, in which the fluorophore moiety is coupled to C-24 of cholesterol. This compound has been used to study cholesterol dynamics in GUVs and plasma membranes (Solanko et al., 2013). BODIPY derivatives have found a niche in membrane fluorescence studies because of their high stability and high quantum yield.

Imidazolium-derivatized lipids constitute a newly introduced family of probes synthesized using click chemistry (Rakers et al., 2018; Bornemann et al., 2020). One such compound is a cholesterol sensor termed CHIM (CHolesterol-based IMidazolium salt) (Matos et al., 2021). The imidazolium group is linked to cholesterol C-2. Using strain-promoted azide-alkyne cycloaddition click bioconjugation chemistry, a fluorophore was added, resulting in CHIM-L. In this sensor, the fluorophore remains outside the membrane, while the rest of the molecule partitions into liquid-ordered (Lo) cholesterol-rich domains (Matos et al., 2021).

Among the fluorescent sensors aimed at characterizing lipid domains in membranes is the fluorescein derivative of cholesterol, fPEG-cholesterol, i.e., cholesterol coupled to a polyethylene oxide [poly (ethyleneglycol)cholesteryl ether]. Introduced by Toshihide Kobayashi (Ishitsuka et al., 2005) this and related compounds exhibit variable length and their large PEG moiety renders the fluorescent molecule impermeable to the membrane. These probes therefore anchor at the plasmalemma and are used in the study of living cells in culture or cell suspension upon application from the aqueous medium (Sato et al., 2004). fPEG-cholesterol partitions exclusively in the outer leaflet of the cell membrane and is preferentially partitioned in the cholesterol-rich Lo lipid phase. After long incubation the probe is slowly internalized following a non-clathrin endocytic pathway (Sato et al., 2004). fPEG-cholesterol has been extensively employed alone (Hullin-Matsuda and Kobayashi, 2007) or together with the sphingomyelin-binding protein lysenin (Hullin-Matsuda and Kobayashi, 2007) to label Lo lipid domains in living cells. Together with Toshihide Kobayashi we employed fPEG-cholesterol to follow the dynamics of cholesterol internalization and to test whether nAChR endocytosis followed the same route. Most of the internalized receptor followed a pathway different from that of fPEG-cholesterol (Kamerbeek et al., 2013), in agreement with our early studies with Satyajit Mayor showing that the receptor utilized a RAC-dependent, clathrin- and dynamin-independent endocytic route (Kumari et al., 2008).

Another methodology to probe the physical state of the membrane and explore the preference of the nAChR protein for different regions of the bilayer is the use of the so-called general polarization (GP) of fluorescence. This technique, introduced by Tiziana Parasassi and Enrico Gratton 30 years ago (Parasassi et al., 1986; Parasassi et al., 1991; Parasassi and Gratton, 1992), initially made use of the fluorescent sensor Laurdan (6-dodecanoyl-2-dimethylamino naphthalene). This fluorescent molecule was designed and synthesized by Gregorio Weber, together with Prodan (6-propionyl-2-dimethylaminonaphthalene), to probe the polarity of bovine serum albumin and myoglobin (Weber and Farris, 1979). The GP of Laurdan essentially senses the dipolar relaxation processes in the immediate environment of the proble, which are manifested in relatively large emission spectral shifts. Laurdan partitions into both Ld and Lo phases. The former phase allows more water molecules to populate the interface region; Laurdan senses the dipolar relaxation of water molecules, affecting its own excited state dipole moment (Weber and Farris, 1979) and resulting in red-shifted emission and lower GP values. Fluorescence emission maxima (∼440 in the gel phase and 490 nm in the liquid-disordered phase) are conserved over a wide range of temperatures (Antollini et al., 1996; Bacalum et al., 2013). In combination with fluorescence correlation spectroscopy (FCS), Laurdan GP was used to detect cell membrane heterogeneity (Sanchez et al., 2012).

The phasor geometric plot method (Jameson et al., 1984) introduced Laurdan into the realm of fluorescence lifetime imaging (FLIM), enabling the visualization of lateral membrane heterogeneities in the fluorescence microscope with the FLIM-phasor combination, first applied to measure the influence of cholesterol content and changes in membrane fluidity and phospholipid order in live cells (Golfetto et al., 2013; Ranjit et al., 2018; Gunther et al., 2021). We made extensive use of Laurdan GP to characterize the physical membrane environment of the nAChR. We introduced the ability of Laurdan to act as a Förster resonance energy transfer (FRET) acceptor of tryptophan emission, using the transmembrane tryptophan residues of the nicotinic receptor as donors, thus being able to interrogate the immediate lipid milieu within Förster distances from the membrane-embedded tryptophan residues in a native membrane environment (Antollini et al., 1996; Antollini and Barrantes, 1998; Antollini and Barrantes, 2002).

**FIGURE 1:**
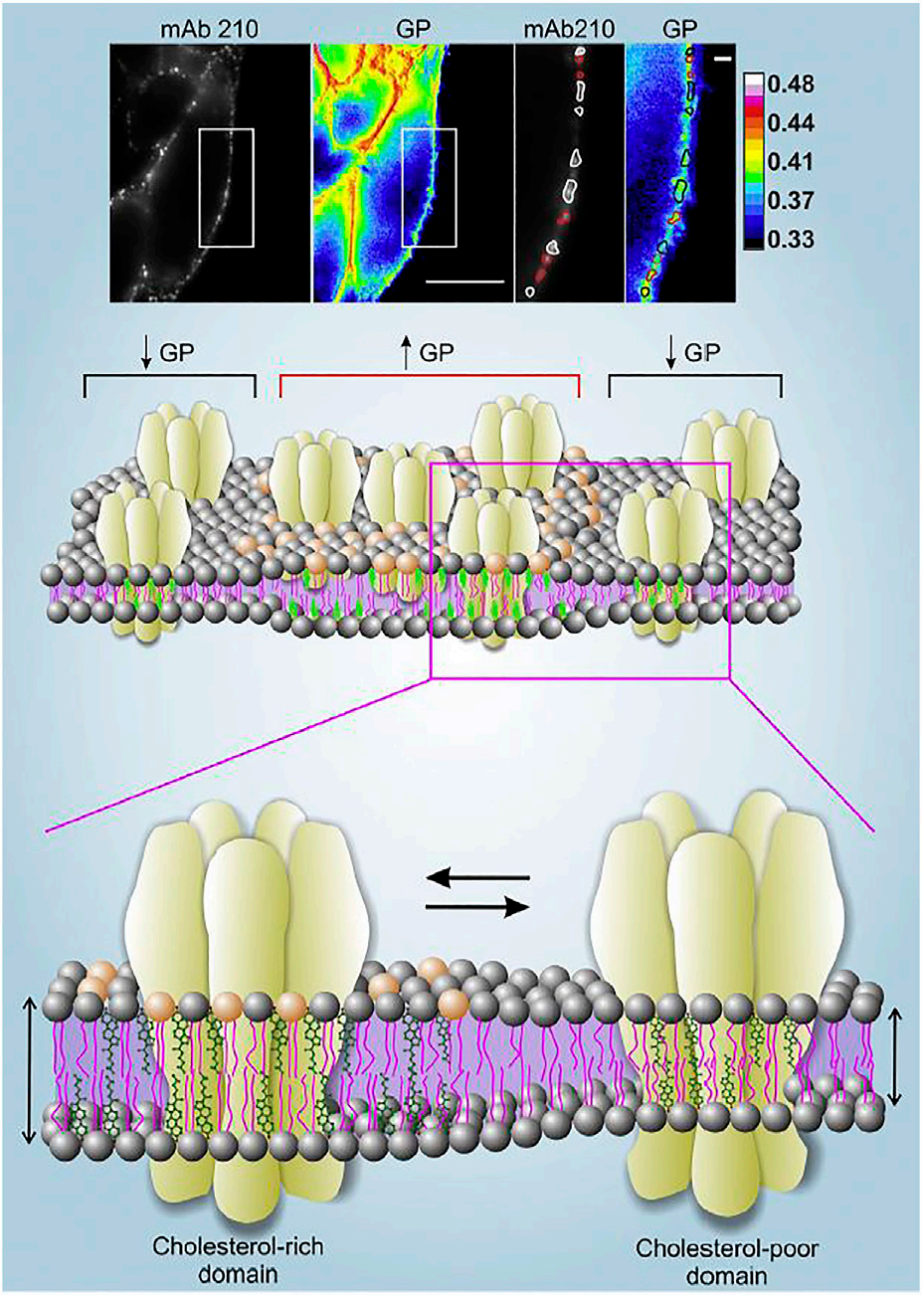
*Upper panel*. Live CHO-K1/A5 cells co-stained with anti-nAChR Alexa647 using the monoclonal antibody mAb210 and the membrane probe di-4-ANEPPDHQ. Regions of the plasma membrane on which the analysis was carried out are highlighted with a white rectangle. nAChR puncta associated with GP values lower than the mean GP of the cell membrane (i.e., Lo domains with lower Chol content) are outlined in white; nAChR clusters with higher-than-the-mean GP are outlined in red. Scale bar: 10 μm in outlined regions of interest, and 1 μm in magnified images. Original data from (Kamerbeek et al., 2013). *Middle and lower panels*. Schematic representation of the distribution of nAChR molecules in liquid-ordered (Lo) and liquid-disordered (Ld) domains rich and poor in cholesterol, respectively. The experimentally determined high GP areas in the upper panel are assumed to correspond to the thicker, cholesterol-rich domains, and vice-versa. Illustration from (Borroni et al., 2016), with permission.

Another environmentally sensitive fluorescent probe is di-4-ANEPPDHQ, a naphthylstyryl-pyridinium (di-n-ANEPPDHQ) compound introduced by Loew and his group (Obaid et al., 2004). Unlike Laurdan, di-4-ANEPPDHQ discriminates between coexisting Lo and Ld phases in model membranes (Jin et al., 2005). The fluorescence emission spectrum of di-4-ANEPPDHQ is blue-shifted ∼60 nm in the Lo phase (max. 610 nm) compared with the Ld phase (max. 560 nm) and exhibits strong second harmonic generation in the Ld phase compared with the Lo phase. The linear combination of the emission maxima can be used in the same manner as with Laurdan GP. To this end we labeled the nAChR in CHO-K1/A5 cells with an anti-muscle type nAChR monoclonal antibody and a secondary antibody tagged with Alexa-Fluor-647 and imaged its cell-surface localization. In parallel, we used a dual-channel recording setup to measure di-4-ANEPPDHQ GP in different regions of the plasma membrane (Figure 1). The nAChR showed a punctiform distribution in both Ld and Lo domains (Kamerbeek et al., 2013). We observed a decrease in di-4-ANEPDHQ GP values upon cholesterol depletion, a change that was correlated with the diminution of nAChR aggregates associated with Lo domains (Figure 1).

### 2.2 Fluorescent probes designed for the cholinergic system and its membrane environment

**FIGURE 2:**
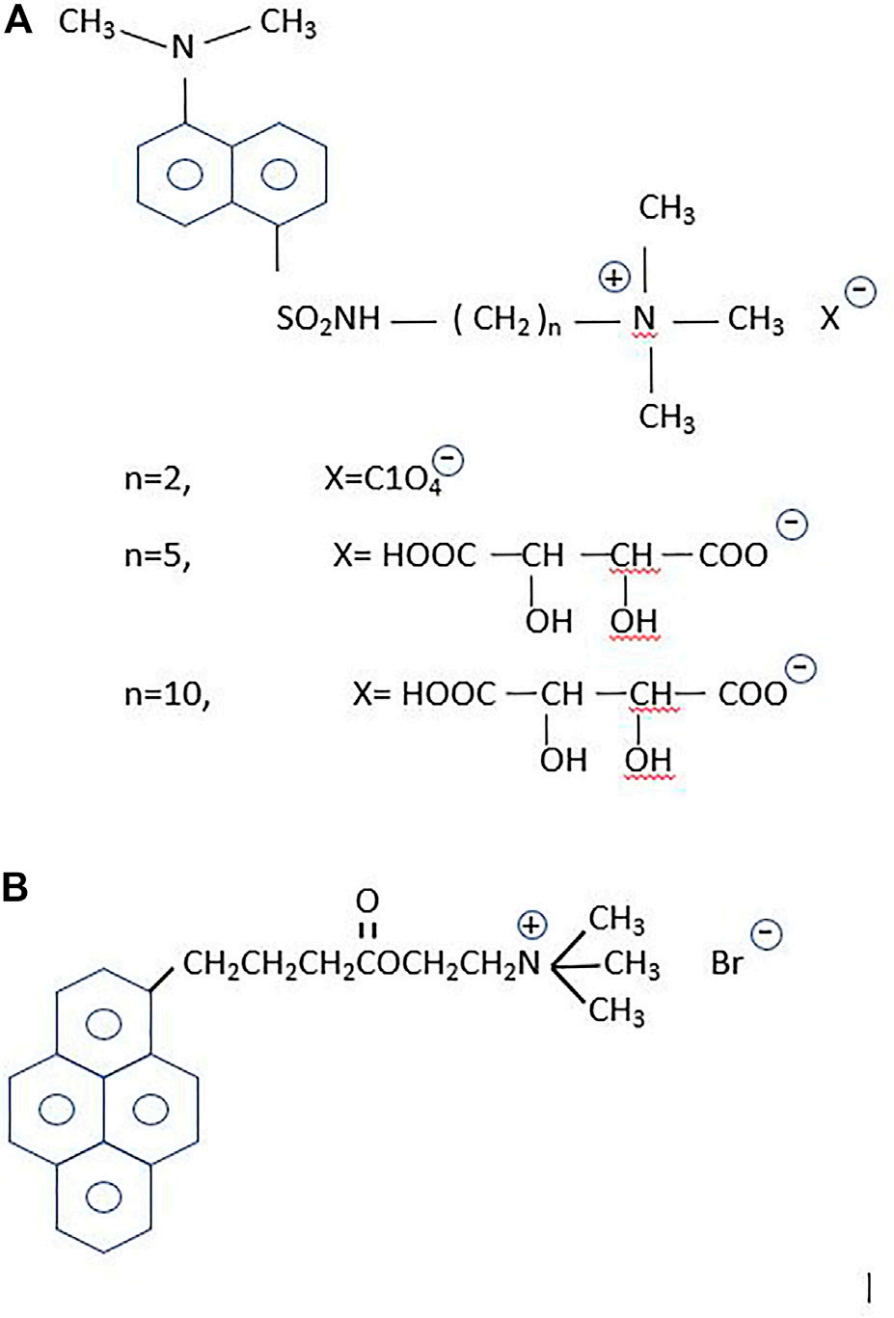
Fluorescent probes for the cholinergic system. **(A)** The dansyl-series. From top to bottom: *n* = 2, the primal compound, dansyl-choline [1-(5-dimethylaminonaphtalene-1-sulfonamido) ethane-2 trimethylammonium perchlorate] (Weber et al., 1971); *n* = 5, [1-(5-dimethylaminonaphtalene1-sulfonamido) pentane-5- trimethylammonium tartrate], and *n* = 10, [1-(5-dimethylaminonaphtalene1-sulfonamido) decane-10-trimethylammonium tartrate] (Narayanan and Balaram, 1978). According to these authors the *n* = 5 and *n* = 10 were intended to increase hydrophobicity and hence enhance the partitioning in lipid membranes of the dansyl homologous compounds, which were employed in binding studies on the enzyme cholinesterase. **(B)** 1-pyrene-butyryl choline, an antagonist of the nAChR (Barrantes et al., 1975) and a high-affinity blocker of the choline uptake mechanism (Dowdall et al., 1976).

Dansyl-choline [1-(5-dimethylaminonaphtalene1-sulfonamido) ethane-2- trimethylammonium perchlorate] constituted the first fluorescent probe designed for the study of a neurotransmitter system (Weber et al., 1971) (Figure 2). Based on this compound, Cohen and Changeux replaced the ethane spacer by a propane group to separate the choline moiety from the fluorophore, and tested its pharmacological properties in the electric organ of *Torpedo marmorata* (Cohen and Changeux, 1973). With the acetylcholinesterases in mind, Narayanan and Balaram subsequently synthesized additional dansyl compounds with the same design but longer spacer arms (Narayanan and Balaram, 1978). Dansyl-choline compounds remain among the cholinergic fluorescent sensors with the smallest fluorophores.

Pyrene is another fluorophore frequently employed by Gregorio Weber to study the dynamics of proteins in solution (Knopp and Weber, 1969). The most characteristic property of pyrene is its exceptionally long fluorescent lifetime. Weber exploited the accessibility of O_2_ and its topography using pyrene as a sensor of surfaces, crevices and the environment (Vaughan and Weber, 1970). The large cholesterol-carrying serum lipoproteins were also ideal targets to label with the pyrene derivative 21-methylpyrenyl-cholesterol (Gaibelet et al., 2013). Our first attempt to exploit the advantageous properties of pyrene as a probe in the study of the nAChR resulted in the synthesis of 1-pyrene-butyryl choline, a long-lifetime compound that delineated the neuromuscular junction and exhibited the electrophysiological and pharmacological properties of an antagonist of the receptor (Barrantes et al., 1975). The probe was also found to be a low nanomolar high-affinity blocker of the choline uptake in cholinergic nerve terminals (Dowdall et al., 1976) (Figure 2).

Another property of pyrene and its derivatives is the ability to form excimers, exploited early in the membrane field to study lateral diffusion coefficients in membranes (Galla and Hartmann, 1980; Somerharju et al., 1985; Dix and Verkman, 1990). The rate of dimer and excimer formation was also exploited to learn about lipid phase diagrams (Feigenson and Buboltz, 2001), lipid domains in membranes, and membrane dynamics (Sassaroli et al., 1990). Pyrene-cholesterol was synthesized to study the lateral distribution of the neutral sterol in the membrane (Lagane et al., 2002). Excimers are formed when a pyrene molecule in the excited state collides with a molecule in the ground state. This depends on both the concentration and lateral diffusion of the probe (Galla and Hartmann, 1980; Holopainen et al., 1997; Somerharju, 2002). We used the excimer formation ability of a pyrene-labeled phosphatidylcholine (Py-PC) to study the effect exerted by the presence of the nAChR protein on the lateral organization of a synthetic lipid membrane. Excimer formation was observed in pure DPPC/DOPC liposomes but was strongly reduced in the presence of the nAChR. We attributed this effect to the restricted diffusion of PyPC in the presence of the receptor protein, which was assumed to rigidify the DPPC-rich regions and increase the apparent concentration of the pyrene sensor in the liquid-ordered domains (Wenz and Barrantes, 2005).

Cysteine residues are favorite amino acid side chains for introducing nitroxide radicals at well-defined locations in membrane proteins studied by electron spin resonance (ESR) spectroscopy. These site-specific tags are then used to learn about the rotational restrictions of the spin label, and hence about the segmental motion of the protein. In a fluorescence study conducted together with Michael Blanton, Manuel Prieto, and Silvia Antollini, we labeled specific cysteine residues in the transmembrane regions of the nAChR with the pyrene sensor N-(1-pyrenyl) maleimide, and applied differential fluorescence quenching with spin-labeled derivatives of fatty acids, phosphatidylcholine, and the steroids cholestane and androstane to locate these residues in the membrane (Barrantes et al., 2000). We applied this strategy to both the intact nAChR molecule purified from *Torpedo californica* electric tissue and to transmembrane peptides obtained by controlled enzymatic digestion from the purified protein. When we analyzed the proteolytic fragments obtained from the intact nAChR, the covalent pyrenyl-maleimide fluorescence mapped to cysteine residues in αM1, αM4, γM1, and γM4. Stern–Volmer plots of the spin-labeled lipid analog quenching showed that stearic acid and androstane spin label derivatives were the most effective quenchers of the pyrene fluorescence. The fatty acid spin-labeled stearic acid 5-SASL isomer quenched more effectively than the 7-SASL and the 12-SASL analogs, indicating a shallow location of the pyrene-labelled Cys residues (Barrantes et al., 2000).

## 3 Breaking the diffraction barrier in light microscopy

Superresolution optical microscopy (nanoscopy) has revolutionized light microscopy, establishing new principles and covering a wide range of applications beyond the biological field: materials science, microbiology, biotechnology, chemistry and physics have benefitted from its inception (Betzig, 2015; Hell, 2015; Moerner, 2015).

The ability to circumvent the diffraction-barrier of light, a physical law formulated by the German physicist Ernst Karl Abbe (Abbe, 1873) has produced a breakthrough in our ability to visualize biological material. As stated in Abbe’s law, the degree of detail that can be resolved by a conventional light microscope is fundamentally limited by diffraction. An infinitely small point source produces a spot of finite volume, the point spread function (PSF), and two such small point sources that are closer together than the half-width of the PSF half-width overlap and are observed as a single object. The resolution in the focal plane can be approximated as 0.5λ/N.A., with λ being the wavelength of light and N.A. the numerical aperture of the objective lens. Using visible light (λ ∼ 550 nm) and a high-N.A. objective lens (N.A. ∼ 1.4) the attainable resolution is ∼200 nm. Superresolution microscopy has found different ways to circumvent this diffraction barrier (Betzig, 2015; Hell, 2015; Moerner et al., 2015). This realization was preceded and later accompanied by major advances in the design and production of new synthetic fluorophores and the discovery of natural fluorescent proteins (Tsien and Miyawaki, 1998; Tsien, 2010) that enabled the application of the new nanoscopies to specific biological research purposes.

Resolution can be improved by restriction of the fluorescence emission to an area much smaller than the PSF. This approach is exploited in the case of stimulated emission depletion (STED) (Rittweger et al., 2007; Sahl and Hell, 2019) and RESOLFT (Hofmann et al., 2005; Testa et al., 2012) nanoscopies. STED is the paradigm direct scanning modality of superresolution optical microscopy whose essential principle is the de-activation of fluorophores in the immediate perimetric volume surrounding the interrogating beam, thereby spatially confining the emission volume (Hell and Wichmann, 1994). Using STED, the nAChR was the first neurotransmitter receptor to be imaged beyond the diffraction barrier, revealing the occurrence of supramolecular aggregates in the form of clusters of nanometric dimensions (Kellner et al., 2007). Subsequent studies from our laboratory have employed total internal reflection fluorescence (TIRF) microscopy and single-particle tracking methods (Almarza et al., 2014) to follow the translational motional regimes of the receptor in live-cell imaging.

## 4 Fluorescence microscopy to study the nanoscale and mesoscale dynamics of nAChRs in the plasmalemma

**FIGURE 3:**
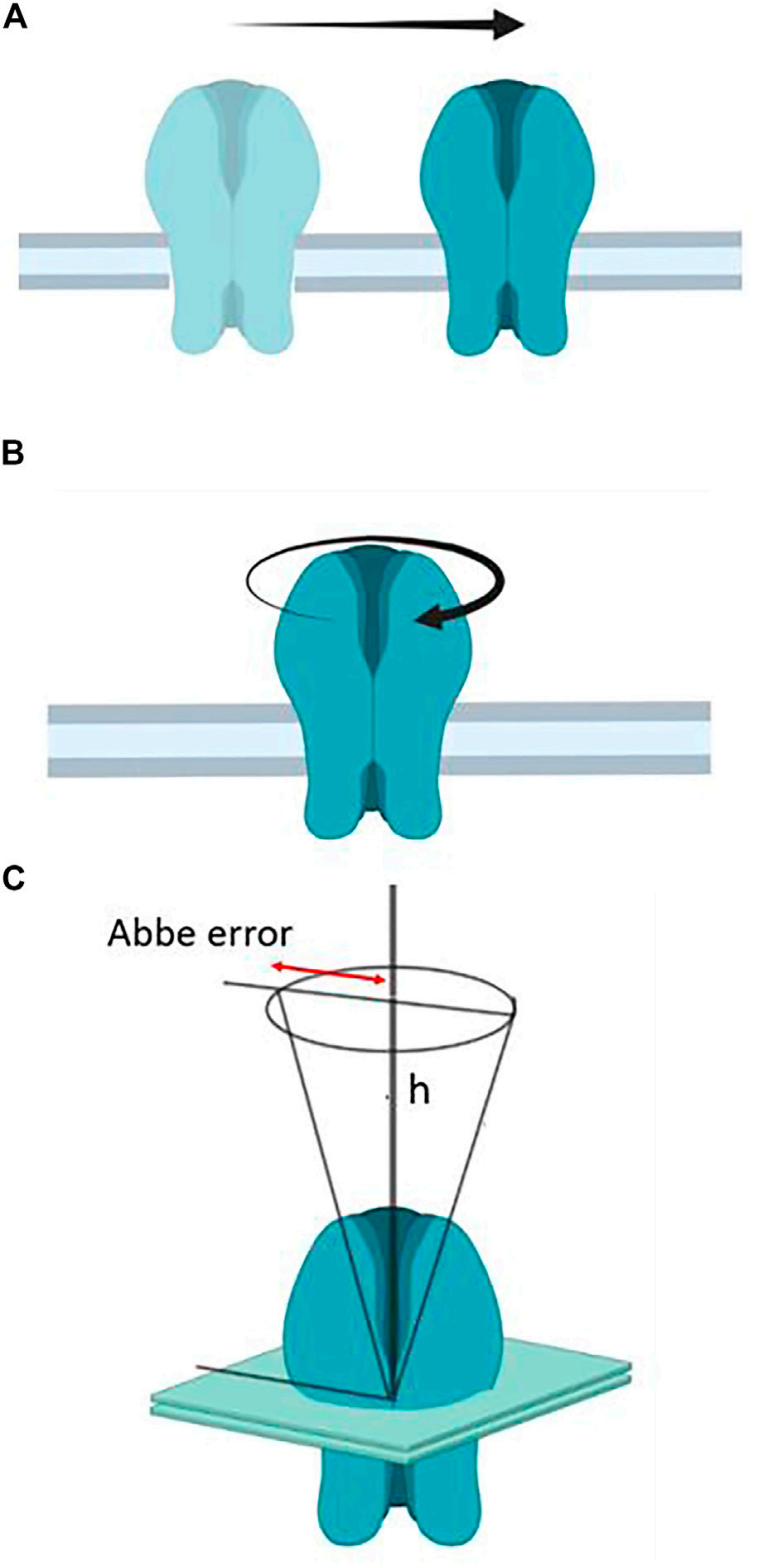
**(A)** Translational or lateral diffusion; **(B)** rotational motion, and **(C)** wobbling motion. In engineering, machine design provides an example of wobbling motion when describing the deviation from parallelism of a spindle axis of a lathe (e.g., when drilling a hole on a plane object). This is the so-called Abbe (again after the German physicist Abbe) or sine error (*ε* = *h* sin *Θ*, where *ε* is the error*, h* the distance, and *Θ* is the angle, here distended by the regular circular cone with its vertex at the center of the nAChR molecule).

As we will discuss in this section, the motions of the receptor from its early embryonic neurodevelopmental stages *in utero* to the mature assembly in the adult neuromuscular junction play key roles in the formation and stabilization of the synapse. Well before nerve endings arrive at the future neuromuscular junction, nAChR molecules diffuse in the plane of the muscle cell membrane to encounter other partner molecules and establish their elementary “socialization”, which will progress to form nanoaggregates, subsequently micron-sized patches of receptors, and upon arrival of the nerve, consolidate the large muscle end-plate. Translational motion is obviously determinant in this ontogenetic process of the peripheral cholinergic synapse, as is the case with the lateral motion of receptors from non-synaptic areas to the synaptic region proper operating in central nervous system synapses that subserve some of the higher brain functions. In addition to the lateral translation of receptor molecules, two other motions can be considered relative to the plane of the membrane: rotational diffusion and wobbling. These two motional regimes have received less attention, and their functional implications are likewise less understood. Figure 3 depicts the three types of motion.

### 4.1 Translational dynamics

In brain synapses, the exchange between surface receptors at the synaptic junction proper and those in non-synaptic areas is considered to underlie neuroplasticity phenomena such as long-term potentiation and depression (Bear and Malenka, 1994; Chapman et al., 2022). These important dynamic phenomena involve the rapid translational motion and subsequent anchoring of glutamate receptors in excitatory synapses [see reviews in (Choquet and Triller, 2003; Triller and Choquet, 2005)]. In the adult peripheral cholinergic synapse, nicotinic receptors are packed into a highly dense platform, the end-plate, with little mobility of individual receptors. Yet lateral mobility of muscle-type receptors is crucial during those developmental stages of synapse formation (Sanes and Lichtman, 2001) when individual molecules start to crosstalk *via* protein-protein interactions. These nanoscale contacts are followed by the tectonic mesoscale lateral motion of receptor clusters to gather into “patches”, which precede, in turn, the assembly of the adult synapse.

During the last half-century, the classical Saffman-Delbrück model has been a dominating theoretical conceptualization of the diffusion of a membrane-embedded body. Based on the classical approach of Brownian motion and the Stokes-Einstein relation, Saffman and Delbrück rationalized the translational and rotational diffusion of membrane-spanning proteins (Saffman and Delbrück, 1975). Translational (lateral) diffusion was conceived to depend, albeit only weaky, on the size (i.e., radius *r*) of the membrane-embedded portion of the protein. Typically, a diffusion process is characterized by the time-averaged mean-square displacement (tMSD), which for a 2-dimensional space like a membrane bilayer can be written as:

$$MSD=<{\Delta r}^{2}\left(t\right)>=\int_{-\infty }^{\infty }r^{2}\left(t\right)P\left(r,t\right)\;d^{2}r=4Dt$$ (1)

Where *D* is the diffusion constant. This assumes a viscous and homogeneous fluid, such that P(r,t) is the probability distribution function (PDF, also termed propagator) of the diffusion process, i.e., the probability of finding the particle at a (radial) distance *r* away from the origin at time *t* after release of the particle at *r* = 0 at time *t* = 0. Complex media may lead to sublinearity of the MSD as a function of time:

$$MSD=<{\Delta r}^{2}\left(t\right)>=K_{\beta }t^{\beta }$$ (2)

In Eq. 2, anomalous diffusion is considered by introduction of the exponent β (Tejedor et al., 2010; Manzo et al., 2015), where $\left(MSD\right)\;∼{\;t}^{\beta }$ . For β=1 simple thermally-driven (“random walk”) Brownian diffusion results. Anomalous diffusion results from other values of β : subdiffusion for 0<β<1 (e.g., in molecular crowding), and superdiffusion for β>1 , usually for molecular motor-driven diffusion. Anomalous diffusion Eq. 2 above can be written in short form as: 4*Dt* ^β^.

The fluorescence recovery after photobleaching (FRAP) technique was introduced in the nAChR field by Daniel Axelrod (Axelrod et al., 1976), who showed that in developing muscle cells, nAChRs aggregated into large (20 μm–60 μm) patches. Receptors in these assemblies are already essentially immobile, with an effective lateral diffusion coefficient (*D*) of < 10^−4^ μm^2^ s^−1^. Lateral mobility of diffusely distributed nAChRs in non-clustered areas of the plasma membrane is ∼ 0.5^−2^ μm^2^ s^−1^ × 10^−2^ μm^2^ s^−1^. Our early attempts to study the lateral motion of the nAChR in the membrane resorted to purified receptor protein samples reconstituted into phospholipid membranes (Criado et al., 1982b). We subsequently combined FRAP with fluorescence correlation spectroscopy (FCS) in the confocal microscopy mode to measure lateral diffusion of the receptor in live mammalian cells (Baier et al., 2010). In our work using muscle-type nAChR heterologously expressed in a mammalian cell line [CHO-K1/A5 (Roccamo et al., 1999)], receptors were labeled with Alexa Fluor 488 α-bungarotoxin. FRAP amounted to only ∼55% of the initial fluorescence 10 min after photobleaching, indicating that ca. half of the receptors were immobile. Cholesterol extraction by methyl-β-cyclodextrin reduced the fraction of mobile receptors from 55 to 20%. Treatment of the cells with the toxin latrunculin A, which induces actin depolymerization, partially restored receptor mobility in cholesterol-depleted cells. In agreement with the FRAP data, the scanning confocal FCS experiments showed that the mobility of the nAChR in small areas of the membrane was about 30% slower upon cholesterol depletion. At that time, we attributed the slower motion of the cholesterol-treated samples to the increase of receptor nanocluster size in cholesterol-poor plasma membranes, as observed with STED nanoscopy (Kellner et al., 2007).

The lateral diffusion coefficient of central nervous system nAChRs is slightly faster than that of the peripheral receptor (McCann et al., 2008; Fernandes et al., 2010). For instance, the lateral mobility of neuronal-type α7 nAChRs in chick ciliary ganglion neurons was found to be 0.070 μm^2^ s^−1^ and 0.188 μm^2^ s^−1^ in synaptic and non-synaptic regions, respectively. Cholesterol extraction from the neurons with methyl-β-cyclodextrin increased the mobility of α7 nAChRs but not that of α3 nAChRs, leading the authors of this study to conclude that neuronal-type nAChR mobility is receptor-subtype specific (Fernandes et al., 2010).

To explore in more detail the effects of cholesterol on nAChR mobility we employed total internal reflection fluorescence (TIRF) microscopy of CHO-K1/A5 cells tagged with Alexa Fluor 488-labeled α-bungarotoxin or anti-receptor antibodies followed by a secondary antibody tagged with the same fluorophore (Almarza et al., 2014). With fluorescent α-bungarotoxin two mobile pools were observed: a highly mobile one undergoing simple Brownian motion (16%) and a second one with restricted motion, amounting to ∼50% of the total. The remaining (∼44%) did not exhibit translational mobility in the time scale of the experiments. We confirmed the conclusions of our previous FRAP/FCS results (Baier et al., 2010) showing that a large proportion of the receptors at the plasma membrane are immobile, while the mobile nAChRs exhibit heterogeneous motional regimes, modulated by the combination of intrinsic (its supramolecular organization) and extrinsic (membrane cholesterol content) factors.

More recently, we applied single-particle tracking techniques in combination with STORM nanoscopy to address the dynamics of individual nAChRs labeled with fluorescent α-bungarotoxin (Mosqueira et al., 2018) and the monoclonal antibody mAb35 (Mosqueira et al., 2020), respectively. mAb35 is one of the monoclonal antibodies produced by Lindstrom and colleagues in a rabbit model; this resulted in the first reproducible experimentally-induced animal model of the human autoimmune disease myasthenia gravis (Tzartos and Lindstrom, 1980; Tzartos et al., 1981). This antibody competes with ∼65% of human antibodies found in myasthenic patients. The behavior of mAb35 is thus presumed to resemble that of the pathogenic autoantibodies found in the human disease. *In vitro*, myasthenia gravis antibodies have been shown to bind divalently to the nAChR, presumably *via* the two α-subunits of adjacent receptor monomers and thus crosslink receptors (see reviews in (Paz and Barrantes, 2019, 2021)). Using mAb35 as a tool to tag *in vitro* cell-surface muscle-type nAChRs, we experimentally observed that the translational motion of the receptor macromolecule upon multivalent ligand (antibody) binding (Mosqueira et al., 2020) differed from the diffusion of the receptor labeled with the monovalent non-crosslinking ligand, fluorescent α-bungarotoxin. Not only did the translational diffusion differ: superresolution microscopy disclosed that the size of the nanoclusters of individual receptor molecules also differed between toxin- and antibody-labeled samples. The implications of these biophysical studies in the pathogenesis of the autoimmune disease myasthenia gravis can be understood in the light of classic observations on the effect of circulating anti-nAChR autoantibodies in myasthenic patients. These receptors crosslink nAChR molecules and augment their internalization (Drachman et al., 1978). In collaboration with Satyajit Mayor in Bangalore, we have reproduced these observations in the mammalian CHO-K1/A5 clonal cell line and in C2C12 developing myotubes; in both cell types mAb35 accelerates the endocytosis of nAChRs (Kumari et al., 2008), thus mimicking the physiopathological hallmarks of the autoantibody-induced receptor internalization in the disease.

In the dynamic studies using the combination of single-particle tracking and STORM nanoscopy, mAb35 crosslinking of cell-surface nAChRs results in a very high proportion (∼80%) of immobile molecules (Mosqueira et al., 2020), whereas using the fluorescent monovalent ligand Alexa Fluor 532 α-bungarotoxin, immobile receptors accounted for ∼ 50% of the total population (Mosqueira et al., 2018). The single-molecule trajectories of antibody-tagged nAChR exhibit longer confinement sojourns (between 340 ms and 440 ms) (Mosqueira et al., 2020) than those of receptors labeled with the non-crosslinking, monovalent ligand α-BTX (135 ms–257 ms) (Mosqueira et al., 2018), indicating that mAb-crosslinked receptors remain for longer periods in crowded areas. At the ensemble level, dynamic clustering is also detected using the approaches developed by Cissé (Cisse et al., 2013; Andrews et al., 2018); these dynamic and transient aggregates occur in areas ∼4-fold larger than those of the confinement sojourns of individual single-molecule trajectories (Mosqueira et al., 2020), and the average lifetime of the antibody-tagged (nano)clustering events was found to be 3-fold longer than those of the corresponding BTX-labeled nAChRs (Mosqueira et al., 2018). These findings are consistent with the notion that mAb35-induced crosslinking adds stability to the nanoclusters (Barrantes, 2021b). Sil and coworkers (Sil et al., 2020) define the nano-scale organization of the CD44 as “being built of individual molecules brought together within ∼10 nm distances”, and meso-scale as “domains ∼100 nm–1,000 nm in scale”. These authors also concur with our results (Mosqueira et al., 2018; Mosqueira et al., 2020) in that the nano-scale behavior of the molecules dictates their meso-scale organization and dynamics, emphasizing the importance of the dynamic nanoclusters in the organization of the peripheral cholinergic synapse (Barrantes, 2007), particularly during the embryonic stages of neuromuscular junction formation. The nAChR nanoclusters likely constitute the intermediate supramolecular organization leading to the meso-scale diffraction-limited “microaggregates” observed during the embryonic stages of neuromuscular development [(Olek et al., 1986a; Olek et al., 1986b) reviewed in (Sanes and Lichtman, 2001)]. During postnatal NMJ ontogeny, these nanoclusters can coalesce to form the micron-sized “patches” and eventually be stabilized by innervation in the form of an adult neuromuscular synapse (Lichtman and Sanes, 2003).

### 4.2 Rotational dynamics

Rotational motion of membrane proteins has received less attention than translational motion. In their classical “hydrodynamic model”, Philip Saffman and Max Delbrück described the translational (*D*_*t*_ *)* and rotational (*D*_*r*_ *)* displacements and the corresponding diffusion coefficients of generic bodies and more realistically, of proteins and lipids in a biological membrane. The thermally-induced rotation θ(*t*) of a transmembrane protein as a function of time *t* can be approximated by that of an ideal membrane-spanning cylinder of radius *R* about its axis perpendicular to an infinite plane sheet of viscous fluid (the membrane) separating infinite regions of less viscous liquid (water) (Saffman and Delbrück, 1975). The current picture of the extracellular medium and the cytoplasmic region is quite different; we now envisage the medium bathing the plasma membrane as a highly crowded mesh of glycolipids and glycoprotein molecules. Similarly, the cytoplasmic region immediately subjacent to the plasma membrane contains a dense cytoskeleton actin meshwork (Goswami et al., 2008; Koster et al., 2016; Koster and Mayor, 2016; Sil et al., 2020).

The angular rotation in time *t* in the plane of the membrane can be represented as:

$$\theta^{2}={2D}_{R^{t}}$$ (3)

As described by Hummer and coworkers in a recent work exploring size corrections for the rotational diffusion coefficients of membrane proteins in molecular dynamics studies (Vögele et al., 2019), for long times *t*, the MSD grows as:

〈(θ(t+t0) – θ(t0))2〉t0≈a+2Dt (4)

for a continuous trajectory of the angle θ(*t*).

The diffusion coefficients of the laterally diffusing particle and the rotating particle are correlated to their mobilities by the Einstein relations:

$$Dt=kB\;Tb_{T}\;and\;D_{R}=kB\;Tb_{R}$$ (5)

Thus the Saffman-Delbrück model predicts a rotational (Eqs 4 and 5) diffusion coefficient for a membrane protein of approximately cylindrical shape to be:

$$D_{R}=\frac{k_{B}T}{4\pi \eta_{m}{R_{H}}^{2}}=\frac{k_{B}T}{4\pi \eta h{R_{H}}^{2}}$$ (6)

where *k*_B_ is the Boltzmann constant, *T* the absolute temperature, $\eta_{m}=\eta h$ is the superficial viscosity of the membrane, *h* its height, and *R*_H_ the hydrodynamic radius of the protein. The Saffman-Delbrück formula is valid for radii *R*_H_ smaller that the Saffman-Delbrück length, η_m_/2 η_f_ where η_f_ is the viscosity of the extracellular medium and the cytoplasm, in the case of the plasma membrane. According to the model, the frictional forces exerted by the biomembrane on the membrane-embedded portion of the protein prevail over other frictional forces. This view remains valid for the periodic Safmann-Delbrück model, an extension of the Safmann-Delbrück original model (Saffman and Delbrück, 1975) that contemplates the effects of periodic boundary conditions on the diffusion constants of lipids and proteins obtained from molecular dynamics simulations (Venable et al., 2017; Vögele et al., 2019).

For most transmembrane proteins the rotational diffusion coefficient *D*_*R*_ lies in the range of microseconds or longer. This is at or beyond the limit of the experimentally-accessible span of most fluorescence techniques like time-resolved or frequency domain fluorescence anisotropy using standard fluorescence probes or even continuous-wave electron paramagnetic resonance (EPR) (ESR) techniques using nitroxide spin labels. The time-resolution capability of EPR can enter the microsecond-to-millisecond time window when using saturation transfer ESR spectroscopy, thus enabling the exploration of the rotational motion of membrane proteins (Thomas et al., 1976; Sahu and Lorigan, 2020).

Because of the inherent time domain covered by phosphorescence spectroscopy, this technique offers the possibility of utilizing the long-lived triplet state induced by the photoselection ability of plane-polarized light (Razi Naqvi et al., 1973). The light source is, in general, flash excitation, as applied to of a triplet probe. One of the sources of phosphorescence in proteins is that arising from the amino acid tryptophan. The signal has been exploited to learn about protein segmental flexibility at room temperature (Mazhul et al., 1999). As a rule, however, the motion of soluble globular proteins in solution is characterized by large amplitudes in the low frequency range, resulting in a marked quenching of their triple state, especially by O_2_. The phosphorescence lifetime of proteins in buffer ranges from (sub) milliseconds for tryptophan residues at solvent-exposed positions to several seconds if the residue lies within a rigid core region of the protein. This determines that phosphorescence is usually detected from the latter set of tryptophan residues at room temperature (Strambini and Gonnelli, 1985). With a size in between O_2_ and acrylamide, the solute acrylonitrile has been shown to penetrate the deep-core, compact regions of various proteins and quench tryptophan phosphorescence (Strambini and Gonnelli, 2010). For reviews on phosphorescence studies of soluble proteins the reader is referred to (Cherry, 1978; Strambini, 1989; Vanderkooi and Berger, 1989).

We studied the rotational mobility of the *Torpedo marmorata* membrane-bound nAChR using phosphorescence anisotropy of eosin-5'-isothiocyanate and eosin-5'-iodoacetamide derivatives of α-bungarotoxin (Bartholdi et al., 1981). Rotational correlation times between 10 μs and 26 μs were measured for the nAChR in dithiothreitol-reduced membrane fragments, compatible with the motion of monomeric receptor species (∼ Mr 260,000), differing from the signal observed in nAChR-rich membranes prepared in N-ethylmaleimide, which contain predominantly the 13-S dimeric species of the receptor (Barrantes, 1982). This difference provided evidence for the sensitivity of the rotational motion to the oligomeric organization of membrane-bound macromolecules.

### 4.3 Tilt of transmembrane segments and wobbling motion

Little is known about the wobbling motion (Figure 3C) of membrane proteins as a whole. Wobbling is certainly present in more localized, segmental motion of proteins. The influence of membrane thickness and lipid composition on the inclination of transmembrane segments relative to the membrane normal has been the subject of numerous investigations. The picturesque term “protein frustration” has been coined in reference to the disparity between the protein hydrophobic thickness and that of surrounding lipids that do not match it, resulting in hydrophobic mismatch (Fattal and Ben-Shaul, 1993; Killian et al., 1996; Nielsen et al., 1998; de Planque and Killian, 2003; Lee, 2003; Marsh, 2008b). The possible mechanisms whereby a membrane protein avoids mismatch has been summarized by Hemminga and coworkers as follows: a) changes in amino acid side chain conformational space; b) changes in backbone conformations; c) changes in tilt angle of the transmembrane segments; d) changes in partitioning in the bilayer; e) changes in aggregation state in the membrane (Stopar et al., 2006). Hydrophobic mismatch may impact on a great variety of membrane phenomena: it may influence the motion of membrane proteins, e.g., translational diffusion (Ramadurai et al., 2010), induce the dimerization of membrane proteins (Marsh, 2008a), alter the composition of lipid domains (Lin et al., 2007), sort proteins into specific domains (Dibble et al., 1993; Milovanovic et al., 2015), induce non-bilayer structures (Killian et al., 1996), or modulate the behavior of mechanosensitive ion channels (Perozo et al., 2002).

Together with Manuel Prieto’s and Tony Watts’ groups we have studied the influence of bilayer thickness on the tilt of the nAChR γ-M4 28-mer transmembrane peptide (de Almeida et al., 2004; de Almeida et al., 2006). Similar studies were conducted with the nAChR α-M1 (de Planque et al., 2004). We combined fluorescence spectroscopy (red-edge excitation shift effect, decay-associated spectra, and time-resolved anisotropy) and NMR spectroscopy and found that the indole moiety of tryptophan 6 in the γ-M4 peptide underwent a rapid wobbling motion, albeit severely restricted in amplitude, possibly due to lateral aggregation of homologous peptides (de Almeida et al., 2006).

The reader is referred to the work of Kawato and Kinoshita for a theoretical treatment of the rotational diffusion of a whole membrane-embedded protein relative to the membrane normal and the restricted wobbling of the entire protein or segments thereof (Kawato and Kinosita Jr, 1981). The authors also derive a wobbling diffusion coefficient and the degree of orientational constraint that can be extended to the independent wobbling of the hydrophylic moiety of the membrane protein.

## 5 Future prospects

### 5.1 Expansion microscopy

There are several new approaches that have not been applied yet to the subject of this review. For instance, sample preparative methods that expand the biological sample isotropically, i.e., with the same expansion factor in all dimensions, and permit nanoscale precision imaging with diffraction-limited conventional optical microscopes instead of specialized super-resolution microscopes. These methods fall under the umbrella of “expansion microscopy” although, as indicated, achievement of superresolution relies on the sample preparation rather than on the imaging procedure. Expansion microscopy works by physically separating fluorescent probes after anchoring them to a free-radical polymerized swellable polyacrylate hydrogel. In its breakthrough introduction, a three-color superresolution imaging with a lateral resolution of ∼70-nm could be achieved in both cultured cells and mouse hippocampus with a conventional confocal microscope (Chen et al., 2015). At early stages, expansion microscopy methods suffered from two pitfalls: i) they failed to retain native proteins in the gel and ii) made use of custom-made synthetic reagents of limited availability. In 2016, Boyden and coworkers introduced a modified technique (retention expansion microscopy) that anchored proteins to the swellable gel and permitted subsequent use of widely available, conventional fluorescently labeled antibodies/streptavidin and/or fluorescent proteins to produce multicolor imaging (Tillberg et al., 2016). Other authors followed in the use of conventional fluorescent probes in expansion microscopy (Chozinski et al., 2016). Organic chemistry has played an important role in providing new polymers with enhanced properties, e.g., non-radical tetrahedron-like monomers that can be iteratively expanded (Gao et al., 2019). Covalently linking strategies permitting the direct tagging of the hydrogel with the targeting molecule and fluorophore at the post-expansion stage have also been applied to cytoskeletal components and lipid membranes (Wen et al., 2020). Other refinements of the hydrogel improved the expansibility of the sample from 4.5x to 20x by an iterative expansion procedure (Chang et al., 2017). Nanorulers can be employed to quantify the swelling factor (Scheible and Tinnefeld, 2018). Most recently, 9x hydrogel-expanded nuclear pore complexes and clathrin-coated pit specimens imaged with conventional wide-field microscopy have reached resolutions of ∼ 30 nm (Li H. et al., 2022).

Expansion microscopy has put conventional wide-field microscopy into the realm of nanoscopy. But what about combining expansion microscopy with the optical capabilities of diffraction-unlimited superresolution imaging? Both STED and STORM microscopies have been blended with the expansion technique. STED + expansion resulted in a 30-fold increase in resolution compared with conventional optical microscopy (10 nm lateral and ∼ 50 nm isotropic) (Gao et al., 2018). In the case of STORM, difficulties in implementing this mix was hampered by fluorophore loss during digestion or denaturation of the specimens, or its subsequent gelation, and the incompatibility of expanded polyelectrolyte hydrogels with photoswitching buffers (Zwettler et al., 2020). The combination with the expansion method afforded a 3-fold resolution enhancement in comparison with plain STORM; in the specific application -imaging of chromosomes- the pre- and post-expansion resolutions were ∼ 48 nm and ∼ 18 nm, respectively (Xu et al., 2019). Imaging of clathrin-coated pits with macromolecular resolution was recently accomplished using the expansion-STORM combination and trifunctional anchors (Shi et al., 2021). The improvements brought about by the above tetra-gels were combined with STORM to image DNA origami with ∼ 11 nm resolution (Lee et al., 2021).

### 5.2 Click, bioorthogonal, clip, snAP, and halo-tag organic chemistry reactions*

As analyzed in the preceding section, the increasing resolving power resulting from expansion microscopy combined with nanoscopies strongly relies on the application of new organic chemistry strategies. The discovery of bioluminescent/fluorescent proteins by Shinomura in the early 60′s [reviewed in (Shinomura, 2005)], their expression in cells (Chalfie et al., 1994), and their exponential applications in fluorescence microscopy (Tsien, 1998; Tsien and Miyawaki, 1998) transformed the field by bringing about the genetically-encoded labeling of target molecules and subcellular structures (Cubitt et al., 1995; Kain et al., 1995; Tsien, 2010). However, the amount of photons that could be extracted in the photophysical cycle of the first-generation fluorescent proteins was relatively poor. Triggered in part by this shortcoming and partly by the needs of superresolution imaging, important developments occurred in the last two decades facilitating the design and customized synthesis of organic compounds with unprecedented precision. Some of these new synthetic procedures are based on the “click chemistry” concept, independently introduced two decades ago by K. Barry Sharpless in the United States and Morten Meldal in Denmark, that enabled the controlled and rapid production of new organic molecules or combinatorial libraries through heteroatom links (C-X-C) (Kolb et al., 2001; Tornøe, 2002). These developments enabled the bioorthogonal click labeling of proteins and subcellular organelles with small fluorophores, some new and some classical compounds reintroduced in the field. By “orthogonal” (perpendicular) reactions, chemists refer to reactions that proceed independently in the same medium without interefering with each other. Carolyn Bertozzi and colleagues provided experimental proof that the azide-alkyne cycloaddition covalent reactions of click chemistry could be applied to label target molecules in living cells, e.g., to map glycans on the cell surface (Agard et al., 2004) and even live animals (Chang et al., 2010). The bioorthogonal approach has also been exploited to address the elusive topic of identifying lipid domains in biological membranes, A tunable orthogonal cholesterol sensor has been developed to simultaneously quantify cholesterol in the two leaflets of the plasma membrane (Liu S. L. et al., 2017).. Another bioorthogonal cholesterol sensor was developed and used in conjunction with superresolution microscopy to image lipid domains of <50 nm diameter in the plasmalemma of live cells (Lorizate et al., 2021).

Similarly the concept of “clip” or “clip-off” chemistry is associated with the ability to cleave covalent bonds and thus “sculpt” or etch the composition of the molecules (Shieh et al., 2021). The latter approaches comprise a variety of techniques to introduce cleavable groups in molecules, labilize linkers, break only selected chemical bonds in the molecular lattice, and tailor the structures to produce new molecules that depart from the original ones in topology or properties, while retaining dimensionality (Yang et al., 2022). SnAP chemistry is usually applied to the synthesis of medium-ring (6-9 membered) saturated N-heterocycles, including bicyclic and spirocyclic structures. The SnAP-Tag approach enables the site-specific coupling of organic fluorophores in live cells using self-labeling proteins (Keppler et al., 2003; Gronemeyer et al., 2006; Lukinavičius et al., 2015; Lukinavičius et al., 2016). Halo-tags are another strategy to covalently label fusion proteins with almost diffusion-limited reaction rate constants (Wilhelm et al., 2021). Interestingly, determination of the crystal structures of a Halo-Tag (HaloTag7) and SnAP-Tag labeled with fluorescent substrates has allowed these authors to determine the differing substrate specificities of the two tags.

Importantly, through these new organic chemistry procedures microscopy has incorporated organic reporter groups with one to two orders of magnitude higher photon fluxes and stability than fluorescent proteins (Fernandez-Suarez and Ting, 2008; Dempsey et al., 2011; Klymchenko, 2017; Erdmann et al., 2019).

Membrane proteins pose additional requirements for efficient site-directed labeling of membrane-embedded regions. A variety of membrane synthetic environments are available for the structural biologists and the biophysicists, ranging from the more artificial zwitterionic detergent micelles, liposomes, lipidic-cubic phase systems and polymersomes to bicelles and nanodiscs, mostly used in solid-state NMR and increasingly being applied in structural cryo-electron microscopy studies of membrane proteins. These surrogate membranes provide the right microenvironment to undertake the requisite organic chemistry, adding speed and specificity to the reactions described in this section.

### 5.3 The new challenges of superresolution microscopy

In 2015 Susan Cox highlighted two challenges of the three main nanoscopy approaches: phototoxicity and the speed with which whole live cells or an ensemble thereof can be imaged, and the tradeoffs in resolution, speed, and ease of implementation (Cox, 2015). The progress since then has been remarkable, increasing resolution to frontiers unimaginable a few years ago. Sauer and colleagues recently summarized the three key factors determining image resolution in SMLM: i) localization precision (statistical scattering of the measured position coordinates), ii) localization accuracy (systematic deviation between the measured and true position) and iii) the labeling density (Helmerich et al., 2022). The first of these factors has been the main focus of efforts for improving image resolution, *via* hardware, e.g., by combining improved structured illumination conditions with single-molecule detection (Kalisvaart et al., 2022; Zhan et al., 2022), DNA-PAINT with STORM (Cnossen et al., 2020), using cryo-SMLM and ad hoc software corrections (Schneider et al., 2020; Hinterer et al., 2022), or *via* computational methods that enhance localization precision through engineering of the PSF or other approaches as applied to a single (Shaw et al., 2022) (Li M. et al., 2022) or multiple fluorophores (colocalization precision) (Koyama-Honda et al., 2005; Donnert et al., 2007; Lemmer et al., 2009; Curthoys et al., 2013; Weisenburger et al., 2014; Georgieva et al., 2016; Willems and MacGillavry, 2022).

Data post-processing *via* deep-learning approaches (Möckl et al., 2019) and high-throuput parallel computing with high-performance clusters (Munro et al., 2019) are increasingly being employed to enhance localization precision in SMLM.

Probes are beginning to pose new challenges, too. The distance between fluorophores constitutes a limiting factor for achieving spatial discrimination below the 10 nm barrier, especially at high labeling densities. This is due to the occurrence of dipole-dipole induced resonance energy transfer between the on- and off-states of fluorophores separated by less than 10 nm, which results in accelerated repopulation of the on-state and fluorescence blinking that reduces localization probabilities in SMLM (Helmerich et al., 2022). These authors have recently introduced a multi-pronged approach combining genetic code expansion with unnatural amino acids, bioorthogonal click labeling with small fluorophores, time-resolved fluorescence detection, and photoswitching fingerprint analysis in SMLM to determine the number of and distance between fluorophores in the sub-10 nm range in cells.

Another limiting factor on the road to improving the resolution of superresolution microscopies is the size of the fluorophore proper. Last-generation nanoscopies like 2-D MINFLUX (Balzarotti et al., 2017; Eilers et al., 2018; Masullo et al., 2021), cryogenic nanoscopy (Furubayashi et al., 2019; Furubayashi et al., 2020), iterative modulation-enhanced SMLM (Kalisvaart et al., 2022), improved structured-illumination microscopies (Chen et al., 2018; Markwirth et al., 2019; Zhanghao et al., 2019; Mangeat et al., 2021; Qiao et al., 2021; Smith et al., 2021; Chen et al., 2022; Hunter et al., 2022; Zhan et al., 2022), new high-resolution DNA-PAINT modalities (Schnitzbauer et al., 2017), 3-D MINFLUX (Gwosch et al., 2020; Grabner et al., 2022; Gwosch et al., 2022) or MINSTED (Weber et al., 2021) are now facing the need to introduce smaller probes to resolve structures at the molecular scale. Successful examples are provided by the recent work in which pyrrolysyl-tRNA synthetase and orthogonal tRNA were matched to introduce clickable amino acids into bacterial and mammalian cell proteins, accomplishing 3-D imaging of β-actin in filopodia with a precision of ∼2 nm (Mihaila et al., 2022).

## 6 Concluding remarks

Currently the structure of a biological membrane can be resolved with optical microscopy at a spatial scale in the order of < 5 nm, i.e., well below the subdiffraction resolution and reaching the macromolecular level. True molecular resolution has classically required electron microscopy, X-ray diffraction, or nuclear magnetic resonance techniques, as a rule using purified molecules in crystalline 3D specimens and increasingly with 2D single-molecule techniques in cryo-EM. Convergence of the emerging methodologies in the field of optical microscopy may soon match the spatial resolution of these structural approaches with the advantage of employing the much milder visible light as the interrogating energy source, thus precluding some of the deleterious effects of high-energy wavelengths on the specimen (Barrantes, 2021a). Cryo-EM has recently achieved true atomic resolution on biological molecules (Nakane et al., 2020; Yip et al., 2020). One can anticipate that optical microscopy will soon reach atomic (Ångström) resolution in fixed specimens, and eventually be able to image the cell-surface of a live cell to disclose the dynamic landscape of the plasma membrane in real time with similar spatial resolution.
